# Head and Neck Cancer

**DOI:** 10.1155/2009/358098

**Published:** 2010-02-04

**Authors:** Amanda Psyrri, Barbara Burtness, Paul M. Harari, Jan Baptist Vermorken, Lisa Licitra, Clarence T. Sasaki

**Affiliations:** ^1^Yale University, USA; ^2^Fox Chase Cancer Center, USA; ^3^University of Wisconsin, USA; ^4^Antwerp University, Belgium; ^5^Instituto Nazionale dei Tumori, Italy

Head and neck cancer is a major cause of morbidity and mortality worldwide. The most common cancer in the head and neck area is head and neck squamous cell carcinoma (HNSCC) which is the 6th most common cause of cancer worldwide. Thyroid cancer accounts for approximately 37000 new cases per year in the United States and 1,600 deaths. The purpose of this special issue is to provide an update on recent advances in the understanding of head and neck tumorigenesis and their implications in clinical practice. 

 The most extensively studied pathway for targeted therapy in HNSCC is the Epidermal Growth Factor Receptor (EGFR) pathway. The EGFR-directed monoclonal antibody, cetuximab, is FDA- and EMA-approved for the treatment of HNSCC. Although the vast majority of HNSCCs contain high EGFR levels, clinical responses to EGFR-targeting therapies have been the modest. Molecular predictors for response to EGFR-targeted therapies in HNSCC are needed. The review by Egloff et al. provides a comprehensive and updated overview of candidate predictive markers in response to EGFR-targeted therapies in HNSCC including Src family kinases and describes recent clinical trials combining Src- and EGFR-targeted therapeutics. Fountzilas et al. analyzed retrospectively 37 patients with locally advanced HNSCC treated with concomitant radiotherapy, weekly cisplatin, and cetuximab for a series of biomarkers (tumor EGFR, MET, ERCC1, and p-53 protein and/or gene expression, MMP9 mRNA) and correlated those with treatment response. MMP9 was the only biomarker tested that appears to be of predictive value in cetuximab-treated patients. Validation of this finding in large independent cohorts is needed before its clinical implementation. 

 Molecular classification is a very important research area since the traditional clinical-pathological factors do not provide accurate prognostic information. The review by Ferrari et al. provides a comprehensive overview of the immunohistochemical expression of biomolecular markers in tongue cancer and their relationships with clinical behavior and prognosis. Pentheroudakis et al. evaluated the prognostic significance of mRNA levels of the EGFR family members HER1-4, the Vascular Endothelial Growth Factors (VEGFs) A, B, C, D, and their receptors VEGFR1, 2, 3 in a small retrospective cohort of HNSCC. The authors reported that high expression of the VEGF-C/VEGFR3 axis in recurrent HNSCC is associated with neck failure (soft tissues/lymph nodes) and inferior survival postrelapse but these findings need to be confirmed in large cohorts. 

 In addition to EGFR pathway, major research efforts concentrate on the identification of other targets for therapy in HNSCC. Akt expression and hyperactivation is a frequent event in HNSCC and strongly correlates with disease progression. Simons et al. explored the hypothesis that the Akt inhibitor, perifosine (PER), combined with inhibitors of glutathione (GSH) and thioredoxin (Trx) metabolism induces cytotoxicity via metabolic oxidative stress in human head and neck cancer (HNSCC) cells. The authors showed that PER induces oxidative stress and clonogenic killing in HNSCC cell lines that is potentiated with inhibitors of GSH and Trx metabolism. These data provide a biochemical rationale for the use of inhibitors of GSH and Trx metabolism in combination with PER in combined modality cancer therapies. 

 Nuclear receptors are implicated in carcinogenesis. Knauer et al. summarize the function, prognostic/ther apeutic value, and, most importantly, ongoing preclinical and clinical studies targeting nuclear receptors in HNSCC. Several lines of evidence support the existence of cancer stem cell subpopulation in solid tumors, including HNSCC. These stem cells account for tumor resistance and aggressive behavior. Chen et al. introduce us to the stem cell concept in HNSCC and its potential application in the treatment of HNSCC patients. The eukaryotic translation initiation factor eIF4E is upregulated in approximately 30% of human cancers including HNSCC and this upregulation correlates with poor prognosis in HNSCC. Culjkovic et al. present the biochemical and molecular properties of the oncogenic potential of eIF4E, the potential strategies for eIF4E targeting in the clinic, and their utility in HNSCC patients. Immunotherapy has been used with limited efficacy in several solid tumors including HNSCC. The comprehensive and updated review by Rapidis et al. summarizes the rationale for immunotherapy in HNSCC and the principal approaches under investigation. 

 Advances in radiotherapy promise to increase cure rates and reduce acute and late morbidity of patients with HNSCC. Nath et al. provide a detailed overview of image-guided radiotherapy in head and neck cancer patients as well as clinical studies analyzing its use in target delineation, patient positioning, and adaptive radiotherapy. 

 Accurate staging of HNSCC is essential for developing therapeutic strategies in patients with HNSCC. Al-Ibraheem et al. provide an updated summary on 18F-FDG PET and PET/CT imaging of head and neck cancer Clinical applications of 18F-FDG PET and PET/CT in head and neck cancer include staging, detection of synchronous 2nd primaries, as well as detection of residual or recurrent disease after completion of treatment. Emerging applications are accurate delineation of the tumor volume for radiotherapy treatment planning, monitoring treatment, and prediction in response to targeted therapies. Sentinel node mapping has emerged as a routine procedure for staging of various malignancies, because it can determine lymph node status more accurately. In the review by Vermeeren et al. the sentinel node procedure and its indications in the head and neck region are presented. The authors also discuss the results of SPECT/CT for sentinel node detection and describe how a portable gamma camera may enable intraoperative real-time imaging with improved sentinel node detection. 

 Advances in molecular biology have offered exciting advances in the treatment of iodine-refractory thyroid cancer. Recent novel and promising findings include additional abnormalities in key pathways associated with thyroid tumorigenesis (RET-Ras-BRAF-MEK, RET-beta-cateinin, TRK-PI3K-AKT, and MDM-p53-PTEN), and gene expression abnormalities. The review by Pinchot et al. provides a comprehensive overview of the vital pathways in Medullary Thyroid Cancer tumorigenesis and focuses on interesting pathways for which targeted drug therapies are currently under development. Patients with multiple recurrences of well-differentiated thyroid carcinoma (WDTC) have significantly worse overall survival compared to those who have ≤1 recurrence of their disease. Holler et al. analyzed retrospectively 31 patients with multiple recurrences of WDTC and found that age >45, stage III/IV disease, distant metastasis, vascular invasion, MACIS score >6, and time to recurrence of <12 months were found to be significant predictors for mortality in this subgroup. 

 We hope that this special issue will inspire interests and new research in the field of head and neck cancer. The development of new targeted therapies the identification of novel predictive and prognostic factors will assist in the development of personalized medicine so that therapy can be tailored and optimized in every patient.

Disclosure: PMH has held laboratory research agreements with industry sponsors developing EGFR inhibitors including Amgen, AstraZeneca, Genentech and ImClone during the last 5 years.

*Amanda Psyrri* *Amanda Psyrri* *Barbara Burtness* *Barbara Burtness* *Paul H. Harari* *Paul H. Harari* *Jan Baptist Vermorken* *Jan Baptist Vermorken* *Lisa Licitra* *Lisa Licitra* *Clarence T. Sasaki* *Clarence T. Sasaki*

